# Supplementary material for: Commercial Potential of the Cyanobacterium Arthrospira maxima: Physiological and Biochemical Traits and the Purification of Phycocyanin
Source: Biology (Basel). 2022 Apr 20;11(5):628. doi: 10.3390/biology11050628 (PMC9138259; doi:10.3390/biology11050628)
Supplement: Supplementary file 1 [file biology-11-00628-s001.zip › biology-1669309-supplementary.pdf]

Table S1. Process for the extraction and purification of phycocyanin from cyanobacteria.

Species	Extraction and purification methods	Concentration	Purity (*A_{PC}/A_{280})	Recovery yield (%)	References
Aphanizomenon flos-aquae	1. $(NH_4)_2SO_4$ precipitation<br>2. Hydroxyapatite chromatography	-	4.78	-	[90]
Aphanizomenon gracile	1. Protease inhibitor<br>2. Sonication<br>3. Ultracentrifugation<br>4. Size exclusion chromatography<br>5. Anion-exchange resin	-	5.3-5.8	-	[91]
Arthronema africanum	1. Freeze/thaw and heated at 30 °C for 1 h in 0.001 M potassium phosphate buffer pH 6.7 and 0.15 M NaCl	200 mg/g	0.87	-	[92]
	1. Rivanol sulphate treatments<br>2. $(NH_4)_2SO_4$ precipitation<br>3. Gel filtration chromatography<br>4. $(NH_4)_2SO_4$ precipitation	-	4.52	55.0	
Arthrospira fusiformis	1. Freeze/thaw and heated at 30 °C for 1 h in 0.001 M potassium phosphate buffer pH 6.7 and 0.15 M NaCl	1.28 mg/mL	0.95	-	[28]
	1. Rivanol sulphate treatments<br>2. $(NH_4)_2SO_4$ precipitation<br>3. Gel filtration chromatography<br>4. $(NH_4)_2SO_4$ precipitation	-	4.30	45.7	
	1. Ammonium sulfate precipitation	0.058 mg/mL	2.06	-	[93]
Arthrospira maxima	1. Aqueous two phase extraction step s<br>2. Ultrafiltration<br>3. (NH4)2SO precipitation	-	3.80	29.5	[94]
Arthrospira platensis	1. 0.05 M sodium phosphate buffer p H 7.0 containing lysis buffer	0.0013 mg/mL	0.97	-	[95]
	1. Chitosan affinity precipitation<br>2. Activated charcoal adsorption<br>3. Aqueous two phase extraction	-	5.10	66.0	[96]
	1. Expanded bed adsorption chromat ography<br>2. Ion exchange chromatography	-	3.64	-	[97]
	1. Three aqueous two phase extractio n steps<br>2. Ultrafiltration	-	4.05	85.0	[98]
	1. Dry biomass mixed with water	3.73 mg/mL	-	-	[99]

Species	Extraction and purification methods	Concentration	Purity ($^{*}A_{PC}/A_{280}$)	Recovery yield (%)	References
	1. Dry biomass mixed with phosphat e buffer pH 7	4.20 mg/mL	-	-	
	1. Agitation in shaker with distilled water	3.68 mg/mL	-	-	
	1. Three aqueous two phase extractio n steps<br>2. Ultrafiltration	-	4.02	78.6	[100]
	1. $(NH_4)_2SO_4$ precipitation steps<br>2. Ion exchange chromatography	-	4.0	-	[101]
	1. $(NH_4)_2SO_4$ precipitation steps<br>2. Ion exchange chromatography	-	5.56	67.0	[102]
	1. Chitosan affinity precipitation<br>2. Activated charcoal adsorption	-	4.30	-	[103]
	1. Expanded bed adsorption chromat ography<br>2. Ion exchange chromatography	-	>4.0	59.0	[104]
	1. Freeze/thaw cycles in 20 mM acetat e buffer containing 50 mM sodium chloride and 0.002 M sodium azide pH 5.10	0.0774 mg/mL	0.75	-	[105]
	1. Heating and shaking with phospha te buffer	2.20 mg/mL	-	-	[106]
	1. Freeze/thaw in distilled water	88 mg/g	0.87	-	[107]
	1. Freeze/thaw with phosphate buffer<br>2. Fraction precipitation (40% saturati on)	9.30 mg/mL	1.92	91.0	
	1. Freeze/thaw with phosphate buffer<br>2. Ion exchange chromatography	9.30 mg/mL	3.70	40.0	[108]
	1. Freeze/thaw with phosphate buffer<br>2. Fractional precipitation and ion exc hange chromatography	9.30 mg/mL	4.33	33.0	
	1. Pulsed electric field with distilled water	159.90 mg/g	-	-	[109]
	1. Hydrophobic interaction membran e chromatography steps	-	4.20	-	[110]

Species	Extraction and purification methods	Concentration	Purity (*A_{PC}/A_{280})	Recovery yield (%)	References
	1. Freeze/thaw cycles in 0.1 phosphat e buffer pH 6.8	73.73 mg/g	0.66	-	[111]
	1. Ammonium sulphate precipitation<br>2. Ultrafiltration<br>3. Fixed-bed ion exchange chromatog raphy<br>4. Ultrafiltration	11.1 mg/mL	5.32	42	[112]
	1. lysozyme enzymatic<br>2. Ultrasonic methods<br>3. Ammonium sulfate precipitation a nd dialysis	0.405-0.422 mg/mL	1.866-2.471	-	[113]
	1. Sonication/ ammonium sulfate pre cipitation and dialyses	0.610 mg/mL	2.90	41	[114]
	1. Liquid biphasic flotation for the pu rification of C-phycocyanin from S pirulina platensis microalga	-	3.49	90.4	[115]
	1. Ultrasound-assisted extraction<br>2. Mixing with PEG 4000 (6%, w/v), p otassium phosphate (15%, w/v), dis tilled water and crude extract<br>3. Phase separation	-	2.337	91.18	[116]
	1. Extraction with water	0.164 mg/mL	1.76	21.1	[117]
	1. Freeze/thaw cycles in 0.1 M sodiu m phosphate buffer pH 7. 0 contain ing 1 mM sodium azide	-	0.8	17.5	[118]
	1. Three $(NH_4)_2SO_4$ precipitation step s<br>2. Ion exchange chromatography	-	4.42	45.6	
	1. Microfiltration steps<br>2. Ultrafiltration	-	1.07	-	[119]
Arthrospira sp.	1. Ion exchange chromatography in e xpanded bed mode	-	1.60	-	[101]
	1. Ion exchange chromatography in fi xed bed mode	-	1.70	-	
	1. Distilled water, sodium phosphate buffer and potassium phosphate b uffer (pH 6.5)<br>2. Ultrasonication	44.24 mg/g	0.34	92	[120]
Calothrix sp.	1. Suspended in 0.1 M phosphate buf fer (pH 7.0) containing EDTA and l ysozyme	0.03 mg/mL	0.4	-	[121]

Species	Extraction and purification methods	Concentration	Purity (*A_{PC}/A_{280})	Recovery yield (%)	References
	1. Ion exchange chromatography	-	2.20	93.0	
	1. Hydrophobic interaction chromato graphy	-	3.50	80.0	
Geitlerinema sp.	1. Ammonium sulfate and dialysis<br>2. Gel filtration<br>3. Ion exchange chromatography	42.17 mg/mL	4.046	-	[122]
Halospirulina sp.	1. Ammonium sulfate and dialysis<br>2. Gel filtration<br>3. Ion exchange chromatography	9.508 mg/mL	8.303	-	
Limnothrix sp.	1. Chitosan, activated charcoal, amm onium sulfate precipitation, anion-exchange chromatography	-	5.26	-	[123]
	1. Extraction with water	0.113 mg/mL	2.14	-	[117]
Lyngbya sp	1. Freeze/thaw cycles in 0.1 M sodiu m phosphate buffer pH 7. 0 contain ing 1 mM sodium azide	-	0.67	3.9	[118]
	1. $(NH_4)_2SO_4$ precipitation steps<br>2. Ion exchange chromatography	-	4.59	36.8	
	1. $(NH_4)_2SO_4$ precipitation steps<br>2. Triton X-100 precipitation<br>3. Gel filtration chromatography<br>4. Ion exchange chromatography	-	5.53	60.2	[124]
Nostoc commune	1. Freeze/thaw cycles in 0.1 M sodiu m phosphate buffer pH 7.0	29.66 mg/g	0.62	-	[125]
Nostoc sp.	1. Ion exchange chromatography<br>2. Aqueous two phase extraction	-	3.55	-	[16]
Oscillatoria okeni	1. Freeze/thaw cycles in 1 M Tris-HCl buffer pH 8	39.93 mg/g	1.65	-	[125]
Oscillatoria quadripunctulata	1. Freeze/thaw cycles in 1 M Tris-Cl b uffer pH 8.1	27.43 mg/mL	0.85	-	[126]
	1. Ion exchange chromatography	-	3.31	44.2	
	1. Size exclusion chromatography	-	2.36	-	

Species	Extraction and purification methods	Concentration	Purity (*A_{PC}/A_{280})	Recovery yield (%)	References
	1. Ammonium sulfate fractionation	-	1.26	-	
Phormidium animale	1. Ammonium sulfate and dialysis<br>2. Gel filtration<br>3. Ion exchange chromatography	3.521 mg/mL	4.196	-	[122]
Phormidium fragile	1. $(NH_4)_2SO_4$ precipitation steps<br>2. Hydrophobic interaction chromatography	-	4.52	62.0	[127]
Phormidium sp.	1. Freeze/thaw cycles in 0.1 M sodium phosphate buffer pH 7.0 containing 1 mM sodium azide	-	0.69	4.1	[118]
	1. $(NH_4)_2SO_4$ precipitation steps<br>2. Ion exchange chromatography	-	4.43	35.2	
Pseudanabaena sp.	1. Extraction with water	0.119 mg/mL	3.10	30.4	[117]
Synechococcus sp.	1. $(NH_4)_2SO_4$ precipitation steps<br>2. Ion exchange chromatography	-	4.03	-	[128]
Thermosynechococcus elongates	1. Ammonium sulphate<br>2. Ion exchange column	-	6.6	-	[129]

*A_{PC}: Absorbance at 615 nm to 620 nm.
The weight units are all based on dry weight.
